# Overweight as a Favorable Clinical Biomarker for Checkpoint Inhibitor Therapy Response in Recurrent Gynecologic Cancer Patients

**DOI:** 10.3390/biom11111700

**Published:** 2021-11-16

**Authors:** Thomas Bartl, Arina Onoprienko, Gerda Hofstetter, Leonhard Müllauer, Nina Poetsch, Thorsten Fuereder, Paul Kofler, Stephan Polterauer, Christoph Grimm

**Affiliations:** 1 Department of Obstetrics and Gynecology, Division of General Gynecology and Gynecologic Oncology, Medical University of Vienna, 1090 Vienna, Austria; thomas.bartl@meduniwien.ac.at (T.B.); arina.onoprienko@meduniwien.ac.at (A.O.); Paul.Kofler@campus.lmu.de (P.K.); christoph.grimm@meduniwien.ac.at (C.G.); 2 Department of Pathology, Medical University of Vienna, 1090 Vienna, Austria; gerda.hofstetter@meduniwien.ac.at (G.H.); leonhard.muellauer@meduniwien.ac.at (L.M.); 3 Department of Biomedical Imaging and Image-Guided Therapy, Medical University of Vienna, 1090 Vienna, Austria; nina.poetsch@meduniwien.ac.at; 4 Department of Medicine I, Division of Oncology, Medical University of Vienna, 1090 Vienna, Austria; thorsten.fuereder@meduniwien.ac.at; 5 Karl Landsteiner Institute for General Gynecology and Experimental Gynecologic Oncology, Karl Landsteiner Society, 3100 St. Poelten, Austria

**Keywords:** immunotherapy, immune checkpoint inhibitor, biomarker, overweight, RECIST

## Abstract

Despite increasing clinical interest in adapting checkpoint inhibitor (CPI) therapies for patients with gynecologic malignancies, no accurate clinical biomarkers to predict therapy response and prognosis are currently available. Therefore, we aimed to assess the predictive and prognostic value of pretherapeutic body mass index (BMI) for recurrent gynecologic cancer patients as previously validated for other solid tumors. We evaluated patients with programmed cell death ligand 1 (PD-L1) positive and, in endometrial cancer, also mismatch repair deficient (MMR) gynecologic malignancies, who received the PD-1 inhibitor pembrolizumab as monotherapy (200 mg fixed-dose q3 w) from 2017 to 2020 (n = 48). Thirty-six patients receiving at least four courses were included in the final analysis. Associations between a BMI increase per 5 kg/m^2^ and overall response rate (ORR; complete + partial response), disease control rate (DCR; ORR + stable disease), progression-free (PFS), and overall survival (OS) were assessed. An elevated BMI was univariately associated with ORR (OR 10.93 [CI 2.39–49.82], *p* = 0.002), DCR (OR 2.19 [CI 0.99–4.83], *p* = 0.048), prolonged PFS (HR 1.54 [CI 1.03–2.34], *p* = 0.038), and OS (HR 1.87 [CI 1.07–3.29], *p* = 0.028). All results could be confirmed in the multivariate analyses. Pretherapeutic BMI therefore appears to be a promising readily available biomarker to identify patients with PD-L1-positive and/or MMR-deficient gynecologic malignancies who could particularly benefit from CPI treatment.

## 1. Introduction

The introduction of checkpoint inhibitors (CPIs) marked a paradigm shift in the therapy of a variety of advanced solid tumors. In recent years, CPIs were successfully implemented as first-line options for many non-gynecologic malignancies, such as malignant melanoma, non-small cell lung cancer (NSCLC), or renal cell cancer. However, the role of CPIs in gynecologic malignancies remains controversial.

While recent negative results of sizeable phase III randomized controlled trials seemingly marked epithelial ovarian cancers as “immunologically cold”, the programmed cell death protein 1 (PD-1) inhibitor pembrolizumab gained FDA approval for both programmed cell death ligand 1 (PD-L1)-positive recurrent cervical cancer and MMR-deficient endometrial cancer after progression to prior systemic therapy [1,2,3,4]. More recently, another PD-1 inhibitor, dostarlimab, was also approved by the FDA for MMR-deficient recurrent endometrial cancer patients [5].

As the last word on potential applications of CPIs in gynecologic malignancies has yet to be spoken, and meaningful prospective trials are still ongoing, the accurate selection of patients who are likely to benefit from CPI therapy remains a primary issue.

One of the most validated approaches of patient selection represents the immunohistochemical assessment of tumor PD-L1 expression levels. Given the rationale that cancer cells actively avoid host immune response by expressing ligands specific to PD-1 receptors of the T-lymphocytes, which results in a downregulation of cell cytotoxic activity, it is assumed that PD-L1-positive tumors are more likely to respond to PD-L1 inhibition than PD-L1-negative tumors. However, response rates to CPIs vary remarkably despite positive tumor PD-L1 expression [6,7]. PD-L1 immunostaining also faces methodologic shortcomings. In addition to a lack of standardization of testing and evaluation, a specialized pathology unit is required for assessment. Further assessments of potential molecular biomarkers of CPI therapy response, such as microsatellite status, tumor mutational load or ARID1A-sequencing, which are currently under investigation or are being adapted for clinical routine, require even more expertise and resources. These limitations reinforce the necessity to define novel and readily available clinical biomarkers of CPI therapy response [8].

Recent studies approached this issue by suggesting pretherapeutic body mass index (BMI) as a predictor of therapy response to PD-L1 inhibition in both metastatic melanoma patients and advanced NSCLC [9,10]. An Italian multicenter trial recently reported a pretherapeutic BMI ≥ 25 kg/m^2^ to be independently predictive for CPI therapy response in a cohort of 976 non-gynecologic cancer patients [11]. 

As CPIs have demonstrated promising results for some gynecologic malignancies, defining cost-effective and readily available biomarkers of therapy response is of utmost clinical interest. As no respective data is available for gynecologic cancer patients to date, the present study aimed to assess the association of a pretherapeutically elevated BMI on both therapy response and survival in a cohort of patients with recurrent gynecologic cancers who received the PD-1 inhibitor pembrolizumab.

## 2. Materials and Methods

The present study was designed as a retrospective exploratory chart review of all consecutive patients with recurrent gynecologic cancers who underwent treatment with the PD-1 inhibitor pembrolizumab at the Division of General Gynecology and Gynecologic Oncology of the Medical University of Vienna between 2017–2020. Pembrolizumab was administered to heavily pretreated, palliative patients for whom no standard therapeutic options remained and who would have been otherwise referred to best supportive care. As pembrolizumab therapy for gynecologic cancers still awaits official approval by the European Medicines Agency, patients considered eligible for CPI treatment by the case-managing gynecologic specialist were referred to our institution’s multidisciplinary immune-oncology tumor board for internal authorization as experimental therapy. Only patients with a positive pretreatment PD-L1 tumor immunostaining result (combined positive score [CPS] ≥1) and an ECOG performance score ≤ 2 were considered eligible for CPI treatment according to our institution’s internal standards; known autoimmune diseases were an exclusion criterion. All included endometrial cancer patients had microsatellite instable disease as indicated by immunostaining of mismatch repair protein expression and further confirmed by molecular microsatellite fragment length analysis as previously described [12].

**Figure 1:**
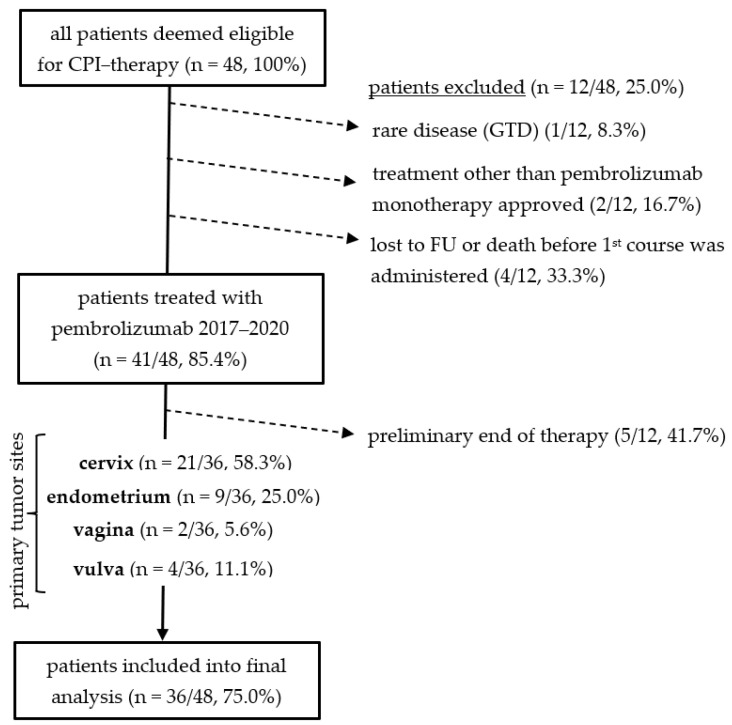
Flowchart depicting the constitution of the patient cohort evaluated at final analysis (n = 36). Out of 48 patients with gynecological cancers initially referred to the immunooncologic tumor board for CPI treatment evaluation, seven patients were excluded for not meeting primary inclusion criteria; another five patients were excluded as they were not re-evaluated after four cycles according to iRECIST criteria for preliminary end of treatment or missing follow-up.

Radiologic assessment according to iRECIST criteria was performed upon approval and after the fourth therapy cycle [13]. Only patients who completed at least four cycles of pembrolizumab monotherapy (200 mg fixed-does q3w) and underwent tumor board response assessment were included for further analysis. Patients with secondary malignant tumors and those who received CPIs other than pembrolizumab were excluded from further analysis. No patient was younger than 18 years (Figure 1).

Therapy responses were classified as overall response rates (ORR, complete + partial response) and DCRs (complete + partial response and stable disease) according to iRECIST assessment after the fourth cycle of pembrolizumab. Overall survival (OS) was calculated as the time between the first administered dose of pembrolizumab and the date of death or last follow-up at our institution. Progression-free-survival (PFS) was calculated as the time between the first administered dose of pembrolizumab and recurrence or progression as documented by the tumor board according to iRECIST assessment. 

As the number of patients in the analyzed cohort was too small to allow for an optimized cut-off assessed by a Youden’s J-statistics, both the BMI per 5 kg/m^2^ increase and at a cut-off of ≥25 kg/m^2^ were adapted as previously proposed [11].

To assess tumor PD-L1 expression, tumor immunostaining was performed on whole tissue sections with a validated anti-PD-L1 antibody (clone BSR90, rabbit monoclonal antibody, Nordic Biosite, Täby, Sweden) in a standardized setting using the ultraView Universal DAB Detection Kit (Ventana Medical Systems, Tucson, AZ, USA) according to staining procedures established at the Department of Pathology of the Medical University of Vienna as previously described [14]. Omission of primary antibody served as negative control. PD-L1 staining results were scored applying the CPS, calculated as the number of PD-L1-positive cells including tumor cells, lymphocytes and macrophages, divided by the total number of viable tumor cells, and multiplied by 100 [15]. Sections were scored individually by two observers, including a pathologist specialized in gyneco-oncology, who were blinded to clinical parameters.

**Figure 2:**
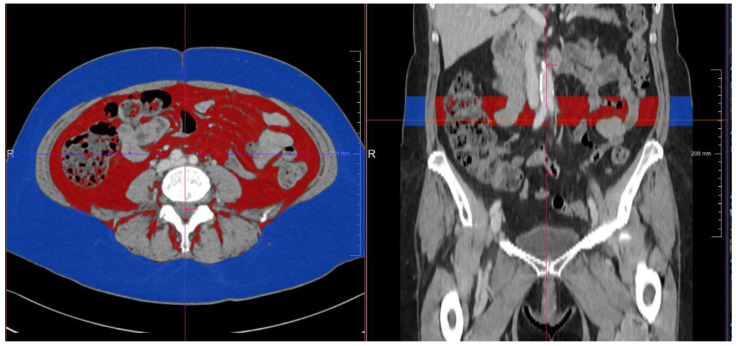
Depiction of computer-tomography (CT)-derived densiometric quantifications of subcutaneous (blue) and visceral (red) fat area performed on image slices between vertebral body L3 and L4. Quantifications were given as volume (mL) and included into statistical analysis in direct comparison to the BMI.

To assess computer-tomography (CT)-derived densiometric quantifications of both subcutaneous and visceral fat area as potential confounders of the BMI, we retrospectively evaluated staging CT-scans, which were routinely performed before CPI-induction. Area-based quantifications were performed on image slices between vertebral body L3 and L4 with a semiautomatic volume tool (Syngo Via, Siemens Healthcare, Munich, Berlin, Germany). By manually marking areas of interest (subcutaneous fat area, visceral fat area), adipose tissue within these areas was selected by limiting measurement thresholds between −150 hounsfield units (HU) and −50 HU as previously described. Results were given as volume (mL) (Figure 2) [16].

Statistical analysis was performed using SPSS^®^ (IBM Corp. Released 2016. IBM SPSS Statistics for Windows, v24.0. Armonk, NY, USA: IBM Corp.) for Windows and R 3.6.3 (R Core Team (2020). R: A language and environment for statistical computing. R Foundation for Statistical Computing, Vienna, Austria. https://www.R-project.org, accessed on 1 June 2021) Categorical variables were described using percentages and medians with interquartile range. A receiver operator characteristic (ROC) was computed to describe the prediction accuracy of the BMI on ORR and DCR.

The BMI per 5 kg/m^2^ increase and clinically relevant confounders (age-adjusted Charlson comorbidity index, CPS, and neutrophile-to-lymphocyte [NLR] ratio calculated as continuous variables) as well as CT-derived visceral and subcutaneous fat volumes were included in univariate logistic regression models to assess associations with ORR and DCR [17]. Respective multivariable logistic regression models were fitted (Section 3.1).

Associations with PFS and OS were assessed fitting cox proportional hazard models both univariately and multivariately (Section 3.3). To graphically depict potential associations of a pretherapeutic BMI ≥ 25 kg/m^2^ with PFS and OS, Kaplan-Meier curves were calculated applying log-rank tests (Section 3.3 and Section 3.4).

CPS was not included into either multivariable analysis as patients were already selected for positive CPS upon inclusion into the study. For all effect estimates, 95% confidence intervals were computed. Two-sided *p*-values < 0.05 were considered statistically significant. The study was conducted in accordance with the Declaration of Helsinki and approved by the ethics committee of the Medical University of Vienna (IRB 1686/2019). 

## 3. Results

### 3.1. Descriptive Characteristics

Between 2017 and 2020, 36 consecutive patients with gynecologic cancers fulfilled inclusion criteria and were evaluated in final analysis. Pretherapeutic baseline patient characteristics are given in Table 1. No differences in pretreatment key characteristics between the cohorts were detected. ORR and DCR broken down by tumor entity were 33.3% (n = 7/21) and 66.7% (n = 14/21) for cervical cancer, 62.5% (n = 5/9) and 75.0% (n = 6/9) for endometrial cancer, and 25.0% (n = 1/4) for vulvar cancer (Table S1). 

PFS and OS were 10.0 months (2.5–17.0) and 14.0 months (6.5–22.0) for cervical cancer, 7.0 months (2.5–11.8) and 7.5 months (3.3–15.3) for endometrial cancer and 3.5 months (2.3–5.5) and 4.5 months (3.3–5.8) for vulvar cancer, respectively. Two patients with vaginal cancer progressed within four cycles of CPI therapy with a PFS of three and four months and an OS of four and ten months. Patients with disease control during CPI therapy (n = 22) experienced significantly longer PFS and OS compared to patients progressing during therapy (PFS 11.0 [5.5–19.0] months vs. 3.0 [2.0–4.8] months, *p* < 0.001; OS 14.0 [6.0–23.3] months vs. 5.5 [3.0–11.8] months, *p* < 0.001).

### 3.2. Predictive Value of a Pretherapeutic BMI for CPI Therapy

Receiver operating characteristics (ROC) demonstrated a classifying ability of pretherapeutic BMI to predict therapy response and disease control with an area under the curve (AUC) of 0.862 and 0.659, respectively (Supplementary Figures S1 and S2). 

The BMI per 5 kg/m^2^ increase was predictive for overall response in both the univariate (OR 10.93 [CI 2.39–49.82], *p* = 0.002) and multivariate analyses (OR 64.09 [CI 1.90–2160.48], *p* = 0.020) (Table 2a). Regarding disease control, BMI was also predictive in univariate analysis (OR 2.19 [CI 0.99–4.83], *p* = 0.048) and multivariate analysis (OR 10.07 [CI 1.33–76.51], *p* = 0.026) as depicted in Table 2b. 

**Figure 3:**
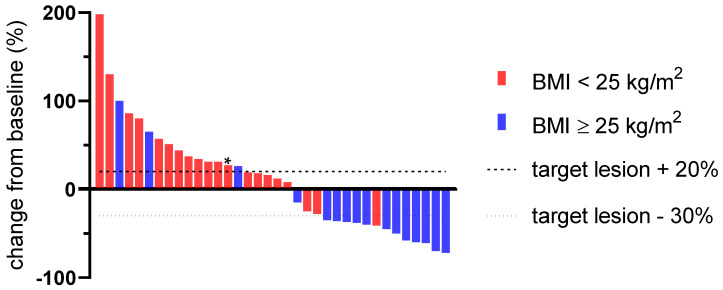
Waterfall plot depicting therapy response assessment after four courses of pembrolizumab according to iRECIST criteria, broken down by pretreatment body mass index. Dashed and dotted lines mark the respective progressive disease and partial response thresholds. The bar marked by an asterisk (*) represents a case which was defined as unconfirmed PD after initial restaging. Progression was not confirmed after eight weeks and was therefore labeled as “stable disease” for further analyses.

Therapy response according to iRECIST criteria and broken down by BMI was further depicted by a waterfall plot in Figure 3. Patients with a pretherapeutic BMI < 25 kg/m^2^ demonstrated a median increase of the target lesion size of 33.8% (1.33 [14.5–62.9]) after four cycles of pembrolizumab as compared to a median decrease of 30.5% (0.70 [0.44–1.21]) in patients with a BMI ≥ 25 kg/m^2^.

### 3.3. Prognostic Value of a Pretherapeutic BMI on Patient Survival during CPI Therapy

A pretherapeutic elevated BMI per 5 kg/m^2^ increase was prognostic for both PFS (HR 1.54 [CI 1.03–2.34], *p* = 0.038) and OS (HR 1.87 [CI 1.07–3.29], *p* = 0.028) in the univariate analyses. Results could be confirmed in the multivariate analyses for PFS (HR 3.73 [CI 1.63–8.50], *p* = 0.002) (Table 3a) and OS (HR 7.44 [1.62–34.16], *p* = 0.010) (Table 3b).

**Figure 4:**
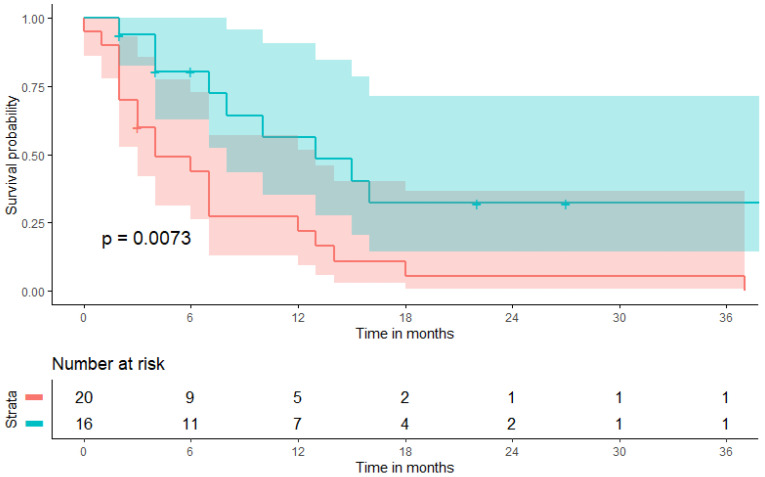
Kaplan-Meier curve depicting progression-free survival (PFS) with confidence interval estimates at the timepoint of pembrolizumab therapy initiation broken down by pretherapeutic body mass index. The blue line depicts the cohort with a pretherapeutic BMI ≥ 25 kg/m^2^, the red line depicts the cohort with a pretherapeutic BMI < 25 kg/m^2^.

**Figure 5:**
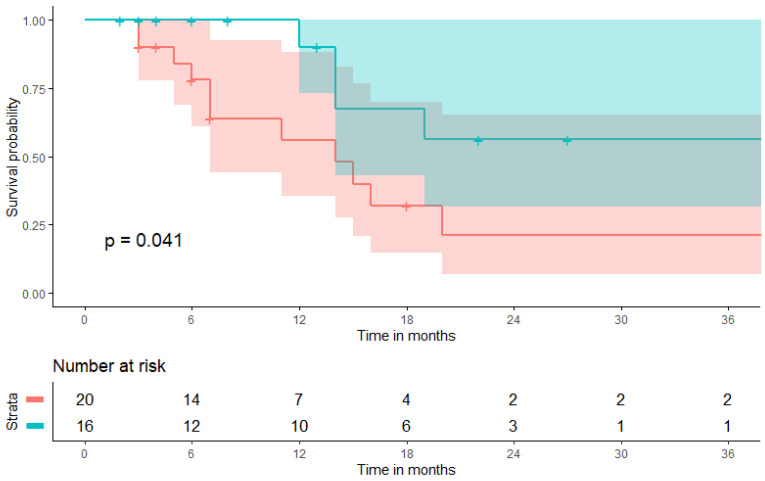
Kaplan-Meier curve depicting overall survival (OS) with confidence interval estimates at the timepoint of pembrolizumab therapy initiation broken down by pretherapeutic body mass index. The blue line depicts the cohort with a pretherapeutic BMI ≥ 25 kg/m^2^, the red line depicts the cohort with a pretherapeutic BMI < 25 kg/m^2^.

Kaplan-Meier curves including confidence intervals graphically depicting the association between a BMI ≥2 5 kg/m^2^ and both PFS and OS are given in Figure 4 and Figure 5, respectively. At a six-months follow-up, 68.8% (n = 11/16) of patients with a pretherapeutic BMI ≥ 25 kg/m^2^ were still receiving ongoing CPI-treatment as compared to 45.0% (n = 9/20) of patients with a pretherapeutic BMI < 25 kg/m^2^. OS remained comparable at a six-months FU with 75.0% (n = 12/16) in the BMI ≥ 25 kg/m^2^-cohort as compared to 70.0% (n = 14/20) in the BMI < 25 kg/m^2^-cohort but diverge at a 12-months FU with 62.5% (n = 10/16) in the BMI ≥ 25 kg/m^2^-cohort as compared to 35.0% (n = 7/20) in the BMI < 25 kg/m^2^-cohort.

### 3.4. Subgroup Analysis Excluding Vaginal Cancer Patients

To account for the slight disbalance in between cohorts regarding vaginal cancer, a subgroup analysis was performed to rule out gross confounding. In the subgroup of all cervical, endometrial, and vulvar cancer patients, excluding vaginal cancer patients (n = 34), associations between a pretherapeutic BMI at a 5 kg/m^2^ increment, ORR (OR 10.66 [CI 2.30–49.38] *p* = 0.002), DCR (OR 2.35 [CI 0.99–5.57], *p* = 0.048), PFS (HR 1.47 [CI 0.98–2.27], *p* = 0.036) and OS (HR 1.89 [CI 1.02–3.52], *p* = 0.044) remained stable in univariate analyses. Results could be reproduced in multivariate analyses for ORR (OR 63.44 [CI 1.86–2167.10], *p* = 0.021), DCR (OR 12.36 [CI 1.26–121.01], *p* = 0.031), PFS (HR 4.27 [CI 1.61–11.13], *p* = 0.004) and OS (HR 4.93 [1.06–1.57], *p* = 0.042) (Supplementary Tables S2 and S3).

### 3.5. Immune-Related Adverse Events (irAEs)

Overall, six cases (16.7%) of irAEs were observed during pembrolizumab therapy. One case of a grade 3 irAE (hepatitis, 2.8%), one case of a grade 2 irAEs (colitis, 2.8%), and four cases of a grade 1 irAEs (thyroiditis, 11.1%) were recorded (Supplementary Table S4). Of note, the grade 1 and 2 irAEs could be managed symptomatically or respective of temporary discontinuation of therapy, but the grade 3 irAE led to permanent discontinuation of CPI therapy after five cycles despite partial response [18].

## 4. Discussion

An elevated pretherapeutic BMI appears to be a positive predictive factor for therapy response to pembrolizumab in patients with recurrent, PD-L1-positive gynecologic cancers. In line, an elevated BMI was associated with improved prognosis in the present cohort. Patients who experienced at least disease control demonstrated a particularly prolonged PFS and OS.

Response rates observed in the present study appear comparable as previously reported for pembrolizumab monotherapies. The KEYNOTE-158 reported an ORR of 57% (CI 42–71) for recurrent MSI-high endometrial cancer patients (n = 49) as compared to 62.5% in the present study [4]. The KEYNOTE-158 also provides the largest sample of PD-L1-positive cervical cancer patients treated with pembrolizumab (n = 82) available to date, describing an ORR of only 14.6% (CI 7.8–24.2) [3]. A retrospective Irish multicenter study, however, demonstrates a remarkably higher ORR of 25%, which appears more in line with present results [19]. More modest response rates of the KEYNOTE-158 may be attributed to a different patient selection, as a disproportionally high number of patients was reported with a FIGO IV stage (93.9% in the KEYNOTE-158 as compared to 19.0% in the present study). 

Evidence on vulvar and vaginal cancer remains particularly scarce. The KEYNOTE-028 reports an ORR of only 5.6% for PD-L1-positive vulvar cancer patients, whereas for vaginal cancer only a case series of two patients has been published to date, of which one responded and one progressed during pembrolizumab treatment [20,21].

The present study is the first to evaluate the predictive and prognostic value of pretreatment BMI in a cohort of gynecologic cancer patients. In contrast to the so-called ‘obesity paradox’ in cancer, a controversially discussed observation of seemingly improved survival of obese cancer patients, the observed phenomenon of both higher response rates and improved survival appears to be specific to CPI treatment [22]. Whereas exact molecular pathological mechanisms are yet to be elucidated, preclinical studies led to the explanatory model that adipogenesis and the overexpression of adipocytokine leptin may modulate the antitumor effects of CPIs [23]. Obesity may also foster a pro-inflammatory state within adipose tissues, resulting in both an increase in total lymphocyte count and an evolution of immune-competent cells to a pro-inflammatory phenotype, which may render them more receptive for immunotherapeutic approaches [24]. 

The association between an elevated BMI and increased response rates to CPI therapy has been broadly validated in the limited spectrum of cancer types, which already experienced broad introduction of CPIs into their treatment algorithms: A recent meta review, which included 13 studies and 5279 cancer patients who have NSCLC, melanoma, or renal cell carcinoma, supports the hypothesis, reporting an elevated BMI to be associated with improved OS and PFS with no significant association to the incidence of irAEs. [25] In a similar vein, a retrospective multicenter study of 976 patients (NSCLC n = 635; melanoma n = 183; renal cell carcinoma n = 135; other n = 23) reported both improved response rates and survival for patients with a pretreatment BMI ≥ 25 kg/m^2^. Patient sex did not significantly influence response rates or survival [11].

As observed, a remarkably strong effect of the pretherapeutic BMI in a small sample of gynecologic cancer patients is in line with recently published sizeable cohorts of other solid tumors. In an era of emerging personalized precision medicine, novel approaches to define reliable biomarkers for therapy response to CPI therapy are urgently needed. The pretherapeutic BMI may thereby represent a promising cost-effective and readily available predictor of respective therapy response and survival, which may find quick adaption in clinical routine after prospective validation.

The present study was, moreover, the first to consider CT-derived quantifications of visceral and subcutaneous abdominal fat volume in comparison to the predictive and prognostic value to a pretherapeutic BMI in gynecologic patients. As the BMI cannot differentiate between different patterns of body fat distribution and thereby identify potential specific fat distribution patterns predictive of CPI therapy response, it was previously hypothesized that CT-derived body fat quantifications might outperform the predictive value of the BMI in this particular question. One study by Young et al. investigating a cohort of 287 metastatic melanoma patients could not observe any predictive value of the BMI and reported only a modest association between very high subcutaneous abdominal fat values and poor survival in female patients. Whereas differing results might be attributed different preconditions in metastatic disease and the different primary, it is to be noted that Young et al. also contradicted a previously published meta-analysis, reporting an improved OS at a BMI ≥ 25 kg/m^2^ for almost 1000 melanoma patients included [26]. No respective evidence is available for NSCLC to date [27].

In the present cohort, subcutaneous body fat demonstrated a significant association with the ORR in univariate analysis; multivariable analysis, however, failed to confirm this observation. Moreover, BMI outperformed its predictive value in direct comparison. As the BMI would also be easier to apply in clinical routine as compared to CT-derived variables, the present study failed to demonstrate any additional diagnostic value of CT-derived quantifications of visceral or subcutaneous abdominal fat. Of note, the present cohort is too small to allow for generalizable assumptions, and further assessments of CT-derived quantifications as a potential biomarker in larger cohorts appear worthwhile.

The present study has some limitations. First, due to its retrospective design, its lack of random patient selection and possible flawed data acquisition alleviates its immediate clinical applicability. Second, as the study design could only include patients with PD-L1-positive tumors, the role of potential reciprocal influence between PD-L1 and BMI remains unanswered. Third, the sample size (n = 36) is too small to allow for generalizable clinical assumptions; the present study was not set out to describe generalizable response rates, but to highlight noteworthy response rates and prognosis differences associated with pretherapeutic patient BMI in recurrent gynecologic cancer patients—an observation in line as previously described for other solid malignant tumors. Presented data is therefore to be considered exploratory and hypothesis-generating and further studies are necessary before potential translation into clinical practice. However, as long as results of prospective trials are to be awaited, collection of retrospective data remains the main pillar to accumulate evidence. To account for potential bias due to unbalanced distribution of pretherapeutic BMI groups across tumor types in a particularly small patient cohort, a subgroup analysis, excluding vaginal cancers, was performed, as both patients with vaginal cancer had a BMI < 25 kg/m^2^. Subgroup analysis results were comparable and did not indicate respective bias.

## 5. Conclusions

BMI appears to be a promising clinical biomarker to predict CPI therapy response and prognosis in patients with PD-L1-positive recurrent gynecologic malignancies. After validation in larger patient cohorts, the BMI may complement established immunohistochemical biomarkers as a readily available and cost-effective stratification factor to improve personalized treatment strategies in the future.

## Figures and Tables

**Table 1:** Descriptive characteristics of patients with recurrent gynecologic malignancies undergoing treatment with the checkpoint inhibitor (CPI) pembrolizumab depicted by body mass index (BMI). Response rates were assessed by restaging after four courses according to iRECIST criteria. Values are given as median (interquartile range) or number (%).

Parameter	All Patients	BMI < 25	BMI ≥ 25	*p*-Value
number of patients	36	20	16	0.679 *
age at CPI induction (years)	56.5 (45.8–65.8)	54.5 (40.8–62.8)	58.5 (52.0–68.3)	0.679 *
CPI courses administered	8 (5–15)	5 (5–10)	10 (7–23)	0.042 *
primary				0.578 ^†^
endometrium	9 (25.0%)	4 (20.0%)	5 (31.3%)	
cervix	21 (58.3%)	12 (60.0%)	9 (56.3%)	
vulva	4 (11.1%)	2 (10.0%)	2 (12.5%)	
vagina	2 (5.6%)	2 (10.0%)	0 (0%)	
body mass index (BMI)	24.7 (20.5–27.6)	21.4 (18.8–24.3)	27.7 (26.3–32.9)	<0.001 *
combined positive score (CPS)	30.0 (5.0–72.5)	35.0 (7.5–84.0)	30.0 (3.5–72.5)	0.639 *
charlson comorbidity index	7 (6–8)	7 (6–8)	7 (7–8)	0.406 *
neutrophile-to-platelet ratio	5.1 (3.6–10.0)	4.9 (3.8–9.4)	9.1 (2.8–11.1)	0.868 *
CT-derived subcutaneous fat volume (mL)	711.8 (334.8–1202.3)	476.7 (223.0–721.7)	1242.9 (904.6–1534.1)	<0.001 *
CT-based visceral fat volume (mL)	327.1 (160.1–557.2)	226.3 (99.5–388.7)	532.4 (285.2–755.4)	<0.001 *
overall response rate (ORR)	36.1% (13/36)	5.0% (1/20)	75.0% (12/16)	<0.001 *
disease control rate (DCR)	58.3% (21/36)	40.0% (8/20)	81.3% (13/16)	0.023 *
progression-free survival (PFS, months)	6.5 (3.0–13.8)	4.0 (2.0–10.8)	9.0 (4.0–20.5)	0.102 *
overall survival (OS, months)	9.5 (4.3–18.8)	7.0 (4.3–15.8)	13.5 (4.5–22.0)	0.499 *

* Student’s *t*-test; ^†^ One-way analysis of variance.

**Table 2:** Univariate and multivariate analysis to assess parameters predictive for overall response (**a**) and disease control (**b**) at the timepoint of pembrolizumab therapy initiation.

**a. Parameters**	**Overall Response after CPI Therapy**			
	**Univariate Analysis**		**Multivariable Analysis**	
	***p*-Value**	**OR (95% CI)**	***p*-Value**	**OR (95% CI)**
combined positive score	0.608	0.99 (0.97–1.02)	-	-
body mass index (5 kg/m^2^ increment)	0.002	10.93 (2.39–49.82)	0.020	64.09 (1.90–2160.48)
neutrophile-to-lymphocyte ratio	0.572	0.95 (0.81–1.13)	0.681	0.94 (0.68–1.28)
age-adjusted charlson comorbidity index	0.373	1.19 (0.81–1.74)	0.418	0.71 (0.31–1.62)
subcutaneous fat volume (100 mL increment)	0.023	1.20 (1.03–1.41)	0.186	0.712 (0.44–1.17)
visceral fat volume (100 mL increment)	0.108	1.26 (0.95–1.66)	-	-
**b. Parameters**	**Disease Control after CPI Therapy**			
	**Univariate Analysis**		**Multivariable Analysis**	
	***p*-Value**	**OR (95% CI)**	***p*-Value**	**OR (95% CI)**
combined positive score	0.163	0.98 (0.96–1.01)		
body mass index (5 kg/m^2^ increment)	0.048	2.19 (0.99–4.83)	0.026	10.07 (1.33–76.51)
neutrophile-to-lymphocyte ratio	0.144	0.89 (0.76–1.04)	0.199	0.87 (0.51–1.40)
age-adjusted charlson comorbidity index	0.968	0.972 (0.241–3.93)	0.506	0.84 (0.51–1.40)
subcutaneous fat volume (100 mL increment)	0.745	1.02 (0.89–1.17)	0.063	0.720 (0.51–1.02)
visceral fat volume (100 mL increment)	0.474	1.10 (0.84–1.44)	-	-

**Table 3:** Univariate and multivariate Cox-regression analysis of parameters prognostic for PFS (**a**) and OS (**b**) at the timepoint of pembrolizumab therapy initiation.

**a. Parameters**	**PFS after CPI Therapy**			
	**Univariate Analysis**		**Multivariable Analysis**	
	***p*-Value**	**OR (95% CI)**	***p*-Value**	**OR (95% CI)**
combined positive score	0.746	1.00 (0.99–1.01)	-	-
body mass index (5 kg/m^2^ increment)	0.038	1.54 (1.03–2.34)	0.002	3.73 (1.63–8.50)
neutrophile-to-lymphocyte ratio	0.767	1.01 (0.95–1.08)	0.789	0.99 (0.93–1.06)
age-adjusted charlson comorbidity index	0.675	1.04 (0.85–1.28)	0.419	1.11 (0.87–1.41)
subcutaneous fat volume (100 mL increment)	0.992	1.00 (0.92–1.08)	0.007	1.23 (1.06–1.43)
visceral fat volume (100 mL increment)	0.487	0.95 (0.82–1.10)	-	-
**b. Parameters**	**OS after CPI Therapy**			
	**Univariate Analysis**		**Multivariable Analysis**	
	***p*-Value**	**OR (95% CI)**	***p*-Value**	**OR (95% CI)**
combined positive score	0.220	1.01 (0.991.–1.03)	-	-
body mass index (5 kg/m^2^ increment)	0.028	1.87 (1.07–3.29)	0.010	7.44 (1.62–34.16)
neutrophile-to-lymphocyte ratio	0.397	1.04 (0.95–1.15)	0.478	1.04 (0.94–1.14)
age-adjusted charlson comorbidity index	0.959	0.99 (0.73–1.36)	0.694	1.07 (0.75–1.53)
subcutaneous fat volume (100 mL increment)	0.620	0.973 (0.873–1.08)	0.035	1.36 (1.02–1.81)
visceral fat volume (100 mL increment)	0.201	0.868 (0.70–1.08)	-	-

## Data Availability

The data presented in this study are available on request from the corresponding author.

## Supplementary Materials

The following are available online at https://www.mdpi.com/article/10.3390/biom11111700/s1, Table S1: Overall response rates and disease control rates of patients with recurrent gynecologic malignancies undergoing treatment with the checkpoint inhibitor (CPI) pembrolizumab broken down by pretherapeutic patient BMI and cancer primary, Table S2: Univariate and multivariate analysis to assess parameters predictive for overall response (S1a) and disease control (S1b) at the timepoint of pembrolizumab therapy initiation in the subgroup excluding vaginal cancer patients (n = 34), Table S3: Univariate and multivariate Cox-regression analysis of parameters prognostic for PFS (S1a) and OS (3b) at the timepoint of pembrolizumab therapy initiation in the subgroup excluding vaginal cancer patients (n = 34), Table S4: Overview of the occurance of documented immune-related adverse events of patients undergoing pembrolizumab monotherapy broken down by BMI.
