# Supplementary material for: Overweight as a Favorable Clinical Biomarker for Checkpoint Inhibitor Therapy Response in Recurrent Gynecologic Cancer Patients
Source: Biomolecules. 2021 Nov 16;11(11):1700. doi: 10.3390/biom11111700 (PMC8615494; doi:10.3390/biom11111700)
Supplement: Supplementary file 1 [file biomolecules-11-01700-s001.zip › biomolecules-1409171-supplementary.pdf]

Supplementary

Table S1. Overall response rates and disease control rates of patients with recurrent gynecologic malignancies undergoing treatment with the checkpoint inhibitor (CPI) pembrolizumab broken down by pretherapeutic patient BMI and cancer primary. Response rates were assessed by restaging after four courses according to iRECIST criteria. Values are given as number (%).

**cancer types by primary**	**overall response rate (ORR)**		**disease control rate (DCR)**	
	**BMI<25**	**BMI≥25**	**BMI<25**	**BMI≥25**
all patients	5.0% (1/20)	75.0% (12/16)	40.0% (8/20)	81.3% (13/16)
endometrium	0% (0/4)	100% (5/5)	25.0% (1/4)	100% (5/5)
cervix	8.3% (1/12)	66.7% (6/9)	58.3% (7/12)	77.8% (7/9)
vulva	0% (0/2)	50.0% (1/2)	0% (0/2)	50.0% (1/2)
vagina	0% (0/2)	-	0% (0/2)	-

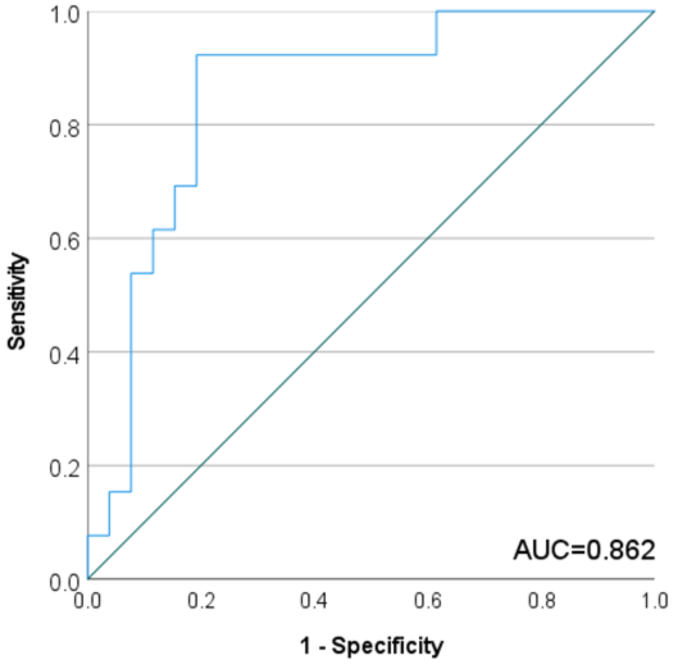

Figure S1. Receiver operating characteristics (ROC) area under the curve (AUC) depicting the classifying ability of a BMI≥25kg/m² to predict overall response after CPI-therapy.

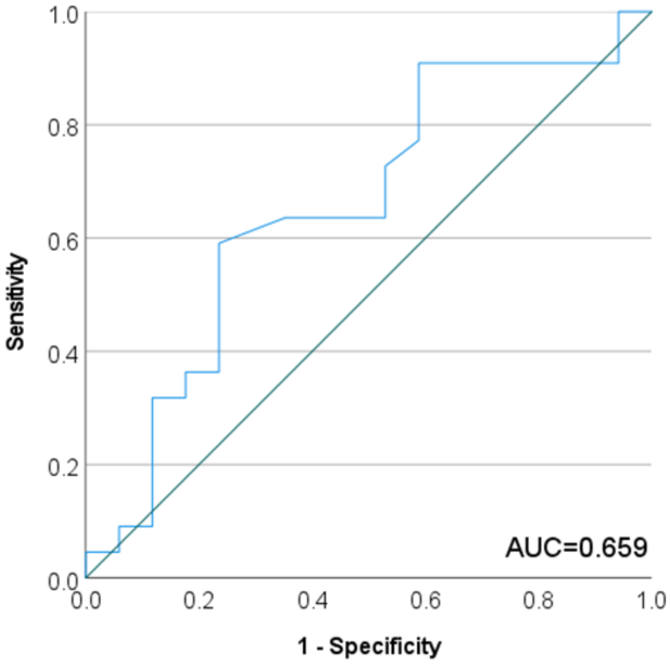

Figure S2. Receiver operating characteristics (ROC) area under the curve (AUC) depicting the classifying ability of a BMI≥25kg/m² to predict disease control after CPI-therapy.

Table S2. Univariate and multivariate analysis to assess parameters predictive for overall response (S1a) and disease control (S1b) at the timepoint of pembrolizumab therapy initiation in the subgroup excluding vaginal cancer patients (n=34).

**S2a. parameters**	**overall response after CPI therapy**			
	**univariate analysis**		**multivariable analysis**	
	**p-value**	**OR (95% CI)**	**p-value**	**OR (95% CI)**
**combined positive score**	**0.568**	0.99 (0.97-1.02)	-	-
body mass index (5 kg/m² increment)	0.002	10.66 (2.30-49.38)	0.021	63.44 (1.86-2167.10)
neutrophile-to-lymphocyte ratio	0.478	0.94 (0.79-1.12)	0.680	0.94 (0.68-1.28)
age-adjusted charlson comorbidity index	0.251	1.26 (0.85-1.89)	0.422	0.71 (0.31-1.63)
subcutaneous fat volume (100ml increment)	0.032	1.19 (1.02-1.39)	0.187	0.72 (0.44-1.17)
visceral fat volume (100ml increment)	0.139	1.23 (0.93-1.63)	-	-

**S2b. parameters**	disease control after CPI therapy			
	**univariate analysis**		**multivariable analysis**	
	**p-value**	**OR (95% CI)**	**p-value**	**OR (95% CI)**
combined positive score	0.274	0.99 (0.96-1.01)	-	-
body mass index (5 kg/m² increment)	0.048	2.35 (0.99-5.57)	0.031	12.36 (1.26-121.01)
neutrophile-to-lymphocyte ratio	0.128	0.88 (0.75-1.04)	0.234	0.86 (0.70-1.09)
age-adjusted charlson comorbidity index	0.803	0.96 (0.66-1.38)	0.392	0.79 (0.46-1.36)
subcutaneous fat volume (100ml increment)	0.636	1.03 (0.90-1.19)	0.082	0.72 (0.50-1.04)
visceral fat volume (100ml increment)	0.395	1.13 (0.86-1.49)	-	-

Table S3. Univariate and multivariate Cox-regression analysis of parameters prognostic for PFS (S1a) and OS (3b) at the timepoint of pembrolizumab therapy initiation in the subgroup excluding vaginal cancer patients (n=34).

**S3a. parameters**	**PFS after CPI therapy**			
	**univariate analysis**		**multivariable analysis**	
	**p-value**	**OR (95% CI)**	**p-value**	**HR (95% CI)**
combined positive score	0.805	1.00 (0.98-1.01)	-	-
body mass index (5 kg/m² increment)	0.036	1.47 (0.98-2.27)	0.004	4.27 (1.61-11.33)
neutrophile-to-lymphocyte ratio	0.630	1.02 (0.95-1.08)	0.789	0.99 (0.92-1.06)
age-adjusted charlson comorbidity index	0.860	1.02 (0.83-1.25)	0.419	1.14 (0.88-1.47)
subcutaneous fat volume (100ml increment)	0.822	0.82 (0.93-1.09)	0.008	1.26 (1.06-1.49)
visceral fat volume (100ml increment)	0.568	0.96 (0.82-1.11)	-	-

**S3b. parameters**	OS after CPI therapy			
	**univariate analysis**		**multivariable analysis**	
	**p-value**	**OR (95% CI)**	**p-value**	**HR (95% CI)**
combined positive score	0.370	1.01 (0.99-1.03)	-	-
body mass index (5 kg/m² increment)	0.044	1.89 (1.02-3.52)	0.042	4.93 (1.06-1.57)
neutrophile-to-lymphocyte ratio	0.239	1.06 (0.96-1.17)	0.448	1.04 (0.94-1.15)
age-adjusted charlson comorbidity index	0.623	0.92 (0.67-1.28)	0.893	1.03 (0.68-1.57)
subcutaneous fat volume (100ml increment)	0.617	1.00 (1.00-1.00)	0.125	1.24 (0.04-1.65)
visceral fat volume (100ml increment)	0.186	1.00 (1.00-1.00)	-	-

Table S4: Overview of the occurance of documented immune-related adverse events of patients undergoing pembrolizumab monotherapy broken down by BMI.

**cancer types by primary**	**occurrence of any type of irAE**			
	**BMI<25**	**type**	**BMI≥25**	**type**
all patients	15.0% (3/20)		18.8% (3/16)	
endometrium	0% (0/4)	-	40.0% (2/5)	one hepatitis (3°),<br>one thyroiditis (1°)
cervix	16.7% (2/12)	one colitis (2°),<br>one thyroiditis (1°)	11.1% (1/9)	one thyroiditis (1°)
vulva	50% (1/2)	one thyroiditis (1°)	0% (0/2)	-
vagina	0% (0/2)	-	-	-
